# Phytic Acid-Iron/Laponite Coatings for Enhanced Flame Retardancy, Antidripping and Mechanical Properties of Flexible Polyurethane Foam

**DOI:** 10.3390/ijms23169145

**Published:** 2022-08-15

**Authors:** Qi Jiang, Ping Li, Yun Liu, Ping Zhu

**Affiliations:** College of Textiles & Clothing, Institute of Functional Textiles and Advanced Materials, National Engineering Research Center for Advanced Fire-Safety Materials D & A (Shandong), State Key Laboratory of Bio-Fibers and Eco-Textiles, Qingdao University, Qingdao 266071, China

**Keywords:** flexible polyurethane foam, phytic acid-iron/laponite, flame retardancy, antidripping, mechanical properties

## Abstract

The use of flexible polyurethane foam (FPUF) is severely limited due to its flammability and dripping, which can easily cause major fire hazards. Therefore, choosing an appropriate flame retardant to solve this problem is an urgent need. A coating was prepared on the FPUF surface by dipping with phytic acid (PA), Fe_2_(SO_4_)_3_·*x*H_2_O, and laponite (LAP). The influence of PA-Fe/LAP coating on FPUF flame-retardant performance was explored by thermal stability, flame retardancy, combustion behavior, and smoke density analysis. FPUF/PA-Fe/LAP has a good performance in the small fire test, which can pass the UL-94 V-0 rating and the limiting oxygen index reaches 24.5%. Meanwhile, the peak heat release rate values and maximum smoke density of FPUF/PA-Fe/LAP are reduced by 38.7% and 38.5% compared with those of neat FPUF. After applying PA-Fe/LAP coating, the value of fire growth rate index decreases from 10.5 kW/(m^2^·s) to 5.1 kW/(m^2^·s), dramatically reducing the fire risk. Encouragingly, the effect of PA-Fe/LAP coating on cyclic compression and permanent deformation is small, which is close to that of neat FPUF. This work provides an effective strategy for making a flame-retardant FPUF with antidripping and keeping mechanical properties.

## 1. Introduction

Flexible polyurethane foam (FPUF), as a kind of 3D open cell material, is widely used in furniture, vehicles, aircraft cushion, and decorative materials, owing to its excellent resilience, permeability, and low density [1,2,3,4]. FPUF plays an indispensable role in modern life [5]. However, due to its composition and structure, FPUF is flammable, with a limiting oxygen index (LOI) of only 18–19% [6]. FPUF quickly releases heat and smoke and starts dripping during the burning process, which can easily cause secondary fire hazards [7,8,9]. Thus, it is urgent to give FPUF flame retardant and antidripping properties.

There are currently two main methods to treat FPUF with flame retardancy: adding flame retardant additives and surface treatment [10,11,12,13]. Nevertheless, adding flame retardant additives in the foaming process inevitably affects the buffering performance, resilience, antideformation, and other mechanical properties of foams, and even leads to unsuccessful foaming [3]. Fortunately, the process of surface treatment is simple and has little impact on the material. Through this method, flame-retardant FPUFs with excellent mechanical properties can be easily and quickly obtained by selecting appropriate flame retardants [14,15]. This method has received extensive attention in recent years.

Halogen flame retardants can effectively inhibit the spread of flames, but halogenated hydrocarbons are produced in the process of combustion, which increases the toxicity of gases and has adverse effects on human health and natural environment [16,17,18,19,20,21,22]. The emphasis on sustainable development has promoted the vigorous development of safe, green, and renewable biomass flame retardants [23,24,25]. In recent years, phytic acid (PA), as a promising biomass flame retardant, has shown outstanding flame-retardant properties [26,27,28]. Meanwhile, PA has a wide range of sources and exists in various plant tissues, such as beans, cereals, and oilseeds [29,30]. When PA is used as a flame retardant, it can act as an acid source to form a complete intumescent flame-retardant system with other carbon sources, and can also cooperate with metals, nitrogen-containing compounds, and other flame retardants [31,32,33]. Recently, PA and sodium lignosulfonate (SLS) have been used to create bio-based coating via dip-coating on polyurethane (PU) sponge [34]. PA1/SLS0.5@PU improves the flame retardancy of PU, preventing melt dripping, self-extinguishing, and reducing peak heat release rate (PHRR) of PU by 32% at a loading level of 60 wt.%. However, the total smoke release (TSR) is not reduced, but increases slightly. Fortunately, both metal ions and inorganic particles can reduce smoke release [35,36,37,38]. Yang et al. [38] use tannic acid (TA)-Fe^III^ MPNs to reduce the total smoke production (TSP) of FPUF to 59.2%. Nonetheless, the TA-Fe^III^ MPNs coated FPUFs cannot achieve self-extinguishing fleetly, and continue to burn for 40 s and remained only very thin char residues after removing the butane igniter. In addition, Nabipour et al. [35] prepared an organic-inorganic hybrid coating with LAP, branched polyethyleneimine, sodium alginate, and chitosan. The coating has a good flame-retardant effect, as the smoke production rate (SPR) value is 61.5% lower than that of neat FPUF, and the coated FPUF self-extinguishes quickly.

In summary, this work aims to prepare a coating with PA, iron (Ⅲ) sulfate hydrate (Fe_2_(SO_4_)_3_·*x*H_2_O), and LAP on FPUF towards improving the flame retardancy and antidripping while maintaining its mechanical properties.

## 2. Results and Discussion

### 2.1. Microstructure and Air Permeability of FPUFs

**Figure 1:**
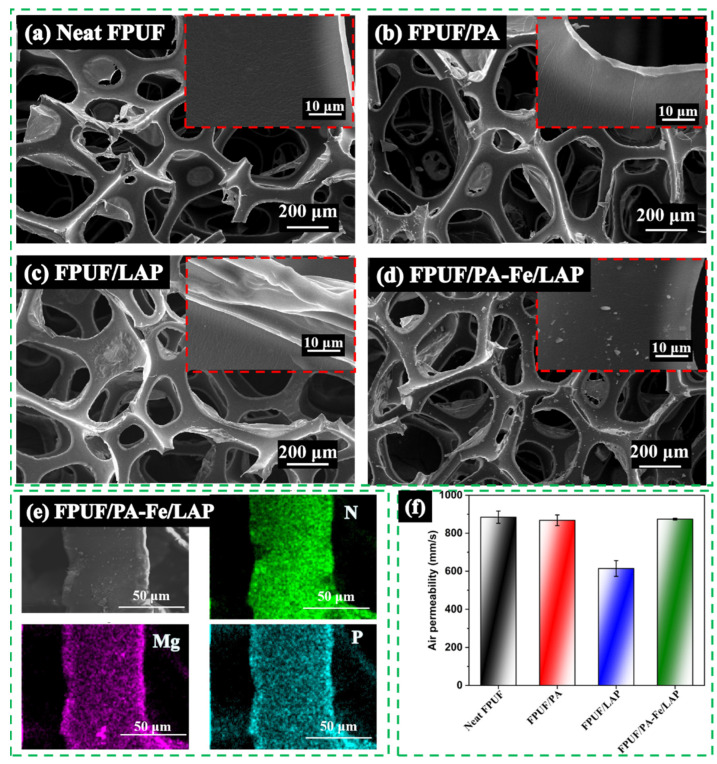
SEM images of neat FPUF (**a**), FPUF/PA (**b**), FPUF/LAP (**c**), and FPUF/PA-Fe/LAP (**d**), EDX mapping images of FPUF/PA-Fe/LAP (**e**), and air permeability of FPUFs (**f**).

The scanning electron microscope with an energy dispersive X-ray analyzer (SEM-EDX) was used to investigate the surface morphology and main element distribution of neat FPUF and flame-retardant FPUFs, as presented in Figure 1a–e. The original cellular structure of FPUF is not changed after flame retardant treatment, which is a major advantage of dip-coating. The coating can be realized without changing the structure. Neat FPUF surface is very smooth at higher magnification, while flame-retardant FPUFs surface is rough, confirming the existence of the coating. Then, the distribution of elements was analyzed by EDX. Mg, P, and N elements are attributed to LAP, PA-Fe, and FPUF, respectively. The elements such as N, Mg, and P are evenly and uniformly distributed as presented in the EDX mapping images of FPUF/PA-Fe/LAP, indicating that PA-Fe and LAP are completely and evenly coated on the FPUF surface.

In addition, except for FPUF/LAP, the air permeability of other flame-retardant FPUFs is close to that of neat FPUF, indicating that the flame retardancy coating does not damage the air permeability of FPUF, as shown in Figure 1f.

### 2.2. Thermal Stabilities

#### 2.2.1. Thermal Stabilities in N_2_

**Figure 2:**
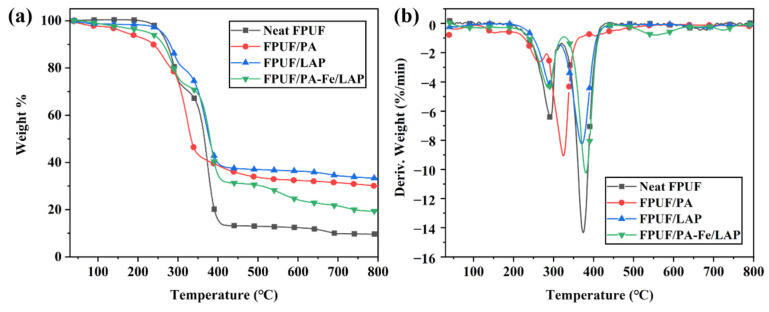
TG (**a**) and DTG (**b**) curves of neat FPUF, FPUF/PA, FPUF/LAP, and FPUF/PA-Fe/LAP in N_2_.

The information related to TG and DTG is shown in Figure 2 and Table 1. The thermal degradation process of neat FPUF under N_2_ is divided into two stages: the rupture of the polyurethane bond at 246–316 °C and the degradation of the polyol hydrocarbon chain at 325–429 °C [39,40,41]. Flame-retardant FPUFs show a similar thermal degradation process. FPUF/PA degrades in advance, as evidenced by the movement of onset degradation temperature (T_5%_) and maximum degradation temperature (T_max_) towards a lower temperature compared with those of neat FPUF. This is mainly due to the low thermal stability of PA, which degrades into phosphoric acid and polyphosphoric acid to promote matrix degradation and char layers formation [29,42]. The existence of protective layers greatly reduces the maximum degradation rate (R_max_) of FPUF/PA compared with neat FPUF, and R_max1_ is reduced from 6.4%/min to 2.6%/min, and R_max2_ is reduced from 14.3%/min to 9.1%/min. LAP, as an inorganic particle, covers the FPUF surface to form a physical barrier, which effectively slows down the degradation rate. PA-Fe/LAP coating not only promotes the formation of char residues due to the degradation of PA to phosphoric acid and polyphosphoric acid, but also owing to LAP, which acts as a physical barrier to hinder the exchange of heat, combustible gases, and oxygen. The Fe ions of PA-Fe/LAP coating can catalyze the formation of more stable char residues [43]. PA-Fe/LAP coating can improve the poorer stability of FPUF/PA in the lower-temperature zone, and the T_5%_ value of FPUF/PA-Fe/LAP increases from 169 °C (FPUF/LAP) to 226 °C. The char residues of FPUF/PA-Fe/LAP at 800 °C are 99.0% higher than that of neat FPUF.

**Figure 3:**
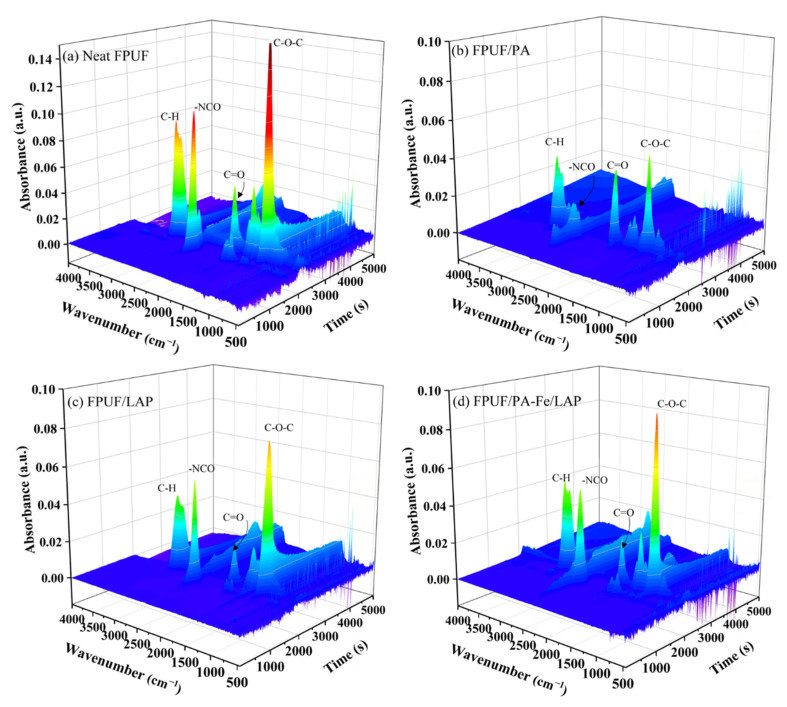
3D FTIR spectra of neat FPUF (**a**), FPUF/PA (**b**), FPUF/LAP (**c**), and FPUF/PA-Fe/LAP (**d**).

**Figure 4:**
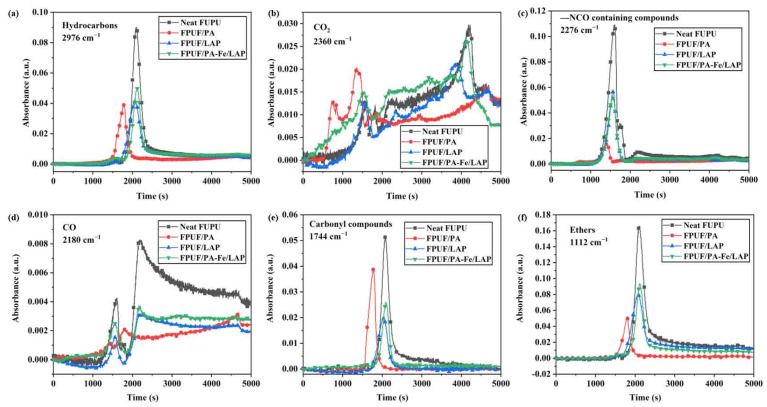
Absorbance intensities of Hydrocarbons (**a**), CO_2_ (**b**), -NCO (**c**), CO (**d**), Carbonyl compounds (**e**) and Ethers (**f**) from neat FPUF, FPUF/PA, FPUF/LAP, and FPUF/PA-Fe/LAP.

To better compare the thermal degradation of neat FPUF and flame-retardant FPUFs, the degradation volatiles of these were determined. The 3D FTIR spectra and FTIR spectra of characteristic substances produced are shown in Figure 3 and Figure 4. The degradation products of neat FPUF and flame-retardant FPUFs are mainly hydrocarbons (2976 cm^−1^), CO_2_ (2360 cm^−1^), -NCO containing compounds (2276 cm^−1^), CO (2180 cm^−1^), carbonyl compounds (1744 cm^−1^), and ethers (1112 cm^−1^) [43,44]. The characteristic peaks of neat FPUF are -CH, -NCO, C=O, and C-O-C. Similarly, for flame-retardant FPUFs, the peaks also appear at the same position, only changing the intensities of the peaks. It shows that the flame-retardant coatings do not change the thermal degradation process of FPUF, but only inhibit the transport of volatile products to the air, leaving more substances in the condensed phase.

As presented in Figure 4, the degradation products of FPUF/PA are released earlier than other samples, which may be caused by the catalysis of phosphoric acid or polyphosphate produced by PA thermal degradation. Nevertheless, FPUF/PA-Fe/LAP does not release volatile products earlier, owing to the physical barrier effect of LAP. The absorption peak intensities of hydrocarbons, carbonyl compounds, and ethers decrease significantly (Figure 4a,e,f), indicating that the released fuels are greatly reduced. In addition, FPUF/PA and FPUF/PA-Fe/LAP release a large amount of CO_2_ in the early stage of thermal degradation; especially FPUF/PA-Fe/LAP begins to release CO_2_ in the first 300 s during the thermal degradation (Figure 4b). The release of CO_2_ is beneficial to dilute the fuels and oxygen concentration. The generation of toxic gases during pyrolysis is also a troublesome problem. However, after flame retardant treatment, the intensities of -NCO and CO absorption peaks decrease relatively (Figure 4c,d), indicating that flame retardant coatings also have a certain effect on reducing the release of pyrolysis toxic volatiles.

#### 2.2.2. Thermal-Oxidation Stabilities in Air

**Figure 5:**
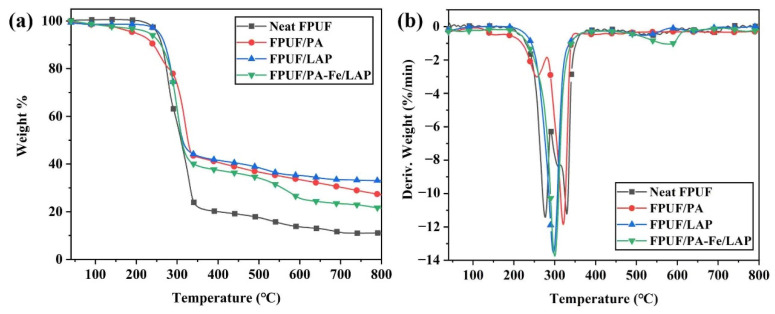
TG (**a**) and DTG (**b**) curves of neat FPUF, FPUF/PA, FPUF/LAP, and FPUF/PA-Fe/LAP in air.

Since the presence or absence of O_2_ may affect the pyrolysis process of materials, the thermal-oxidative degradation process of neat FPUF and flame-retardant FPUFs is also studied as shown in Figure 5 and Table 2. The changing trend of T_5%_ of flame-retardant FPUFs is consistent with that in N_2_. It is worth noting that FPUF/LAP and FPUF/PA-Fe/LAP have only one pyrolysis stage, which can be owing to the presence of LAP as an inorganic clay and its better physical barrier function [35]. It delays the first decomposition stage, resulting in the coexistence of the two degradation stages on the TG curves. Moreover, the amount of char residues increases significantly at 800 °C after flame retardant treatment. The amount of char residues of FPUF/PA, FPUF/LAP, and FPUF/PA-Fe/LAP increases by 144%, 198%, and 102%, respectively, compared with neat FPUF. It indicates that the coatings improve the char formation of FPUF at high temperatures. It is worth noting that FPUF/PA-Fe/LAP has more char residues in air than in N_2_, probably because iron is present as Fe_3_O_4_ during thermal degradation process in N_2_, which can be further oxidized to Fe_2_O_3_ in air [45].

### 2.3. Fire Safety

#### 2.3.1. Flame Retardancy

**Figure 6:**
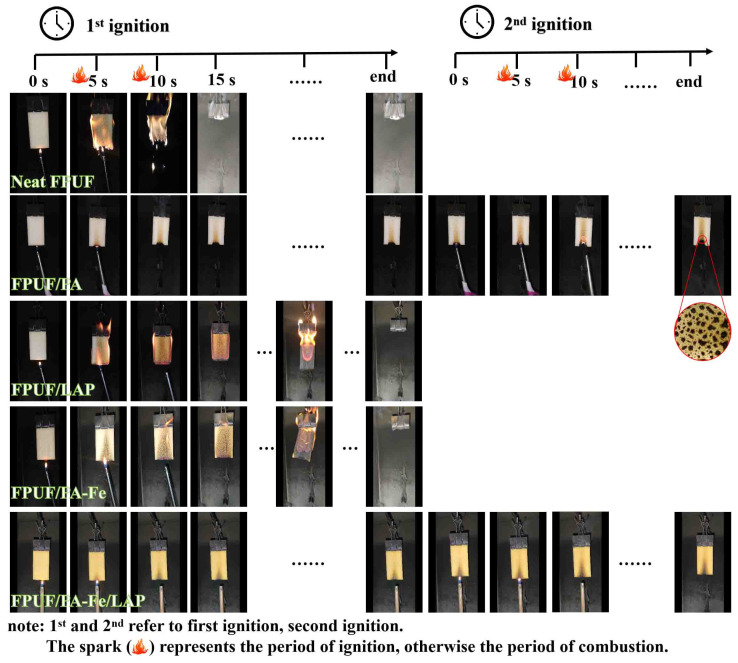
Digital images of neat FPUF, FPUF/PA, FPUF/LAP, FPUF/PA-Fe, and FPUF/PA-Fe/LAP during UL-94.

The vertical flame test (UL-94) and LOI are commonly used to evaluate the flammability of materials [46,47,48,49]. As shown in Figure 6 and Table 3, neat FPUF is a flammable material that burns rapidly upon ignition, and has a low LOI value of only 17.0%. When it is in contact with a flame, neat FPUF spreads rapidly, burns violently, and produces dripping. After flame retardant treatment, the dripping phenomenon has been significantly inhibited to reduce the risk of a secondary fire. LAP coating cannot inhibit the flame spread, while PA-Fe coating can effectively slow down the flame spread. This may be caused by the fact that LAP can only form loose char residues, while phosphoric acid or polyphosphate and metal ions can promote the formation of dense char residues and hinder the transfer of fuels, oxygen, and heat, to slow down the spread of a flame [47]. FPUF/PA can realize self-extinguishing quickly, but PA cannot inhibit the melting of FPUF. There are only small spherical char residues on the FPUF/PA surface, and it is also blackened in the upper part of the sample, which does not have a UL-94 rating. Fortunately, FPUF/PA-Fe/LAP can pass the UL-94 V-0 rating, and the LOI value increase to 24.5%. This could be due to the cooperation between PA-Fe and LAP. PA-Fe has the ability of catalytic char formation and LAP has the ability of physical barrier, which is consistent with the study of Yang [50]. In conclusion, PA-Fe/LAP coating can greatly reduce the flammability of FPUF.

#### 2.3.2. Burning Behavior

**Figure 7:**
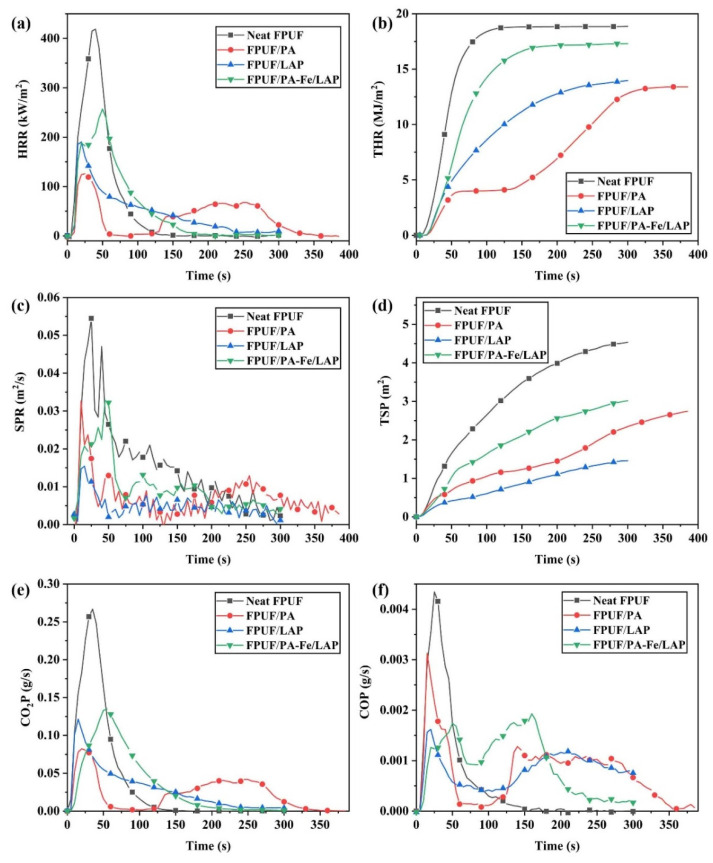
The HRR (**a**), THR (**b**), SPR (**c**), TSP (**d**), CO_2_P (**e**), and COP (**f**) curves for neat FPUF, FPUF/PA, FPUF/LAP, and FPUF/PA-Fe/LAP by CCT.

The burning behavior of neat FPUF and flame-retardant FPUFs in a fire is simulated by cone calorimeter test (CCT), as summarized in Figure 7 and Table 4. The PHRR and total heat release (THR) values of neat FPUF reach 419 kW/m^2^ and 19 MJ/m^2^. The PHRR values of FPUF/PA and FPUF/LAP are reduced by 69.9% and 54.7%, while the THR values of FPUF/PA and FPUF/LAP are decreased by 31.6% and 26.3%. Unfortunately, after coated with PA-Fe/LAP, the PHRR and THR values are only decreased by 38.7% and 10.5%. This is in contradiction with previous studies that metal compounds are used as flame retardants to produce metal oxides to absorb a lot of heat and reduce heat release [32]. This might be due to the metal ions further promoting the degradation of unstable char residues, which reduce the amount of char residues and cannot effectively prevent heat transfer [51]. This is confirmed by the fact that the amount of char residues for FPUF/PA-Fe/LAP is less than those of FPUF/PA and FPUF/LAP. In addition, the time to ignition (TTI) value and T_PHRR_ of FPUF/PA-Fe/LAP are delayed, indicating that it takes longer to be ignited and it can leave more time for people to escape. The ratio of PHRR to T_PHRR_ is equal to the fire growth rate index (FIGRA), which is generally proportional to the fire hazard [42,52]. Notably, the FIGRA values of FPUF/PA (5.0 kW/(m^2^·s)) and FPUF/PA-Fe/LAP (5.1 kW/(m^2^·s)) are drastically reduced, compared with that of neat FPUF (10.5 kW/(m^2^·s)). This indicates that these two coatings reduce the fire hazard.

Smoke is another major factor threatening people’s safety in a fire. The TSP value and peak SPR (PSPR) value of neat FPUF are 4.5 m^2^ and 0.05 m^2^/s. Compared with neat FPUF, the TSP values of FPUF/PA, FPUF/LAP, and FPUF/PA-Fe/LAP are decreased by 40.0%, 66.7%, and 33.3%, respectively. Additionally, the SPR value of FPUF/PA-Fe/LAP increases sharply after 40 s of burning and reaches the PSPR after 50 s, meaning that the risk of suffocation is reduced and people have more time to escape. Moreover, the peak specific extinction area (PSEA) of flame-retardant FPUFs is also reduced, and this indicates that the flame-retardant coatings reduce the production of flammable volatiles. The CO_2_ emission of flame-retardant FPUFs is greatly reduced, leaving more char in the condensed phase. Moreover, the release of CO from flame-retardant FPUFs is relatively lower than that of neat FPUF; especially, the peak CO production rate (PCOP) value of FPUF/LAP is decreased by 62.7%, and the PCOP value of FPUF/PA-Fe/LAP is decreased by 60.0%, which reduce the risk of a fire. The value of the CO/CO_2_ ratio is applied to assess the level of complete burning. The lower the value is, the more complete the burning is. After the flame-retardant treatment, the value of the CO/CO_2_ ratio increases significantly, and this shows that the smoke toxicity increases. Fortunately, the synergistic effect between PA-Fe and LAP can effectively inhibit smoke toxicity. The CO/CO_2_ ratio values of FPUF/PA and FPUF/LAP increase to 0.0581 and 0.0923, respectively, while that of FPUF/PA-Fe/LAP is only 0.0171.

**Figure 8:**
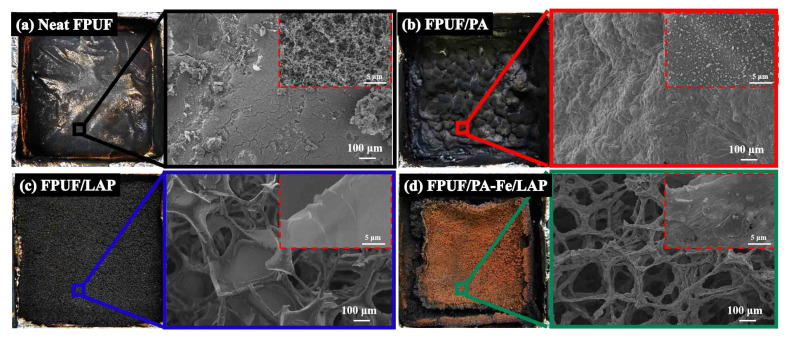
Morphology of the char residues of Neat FPUF (**a**), FPUF/PA (**b**), FPUF/LAP (**c**), and FPUF/PA-Fe/LAP (**d**) after CCT.

Figure 8 presents the information of char residues after CCT. Neat FPUF only leaves a thin layer of char. In the SEM images, there are many cracks on the char residues surface, and the char residues are loose with a large number of holes. Flame-retardant FPUFs can form dense char residues which can effectively protect the matrix. However, PA cannot inhibit the melting of FPUF, and the original cell structure disappears completely. LAP and PA-Fe/LAP can inhibit melting and avoid liquid pool fire.

In a word, the flame-retardant coating can form dense char residues, protect the lower substrate, obviously reduce heat and smoke release, and improve people’s survival rate in a fire.

#### 2.3.3. Smoke Density

**Figure 9:**
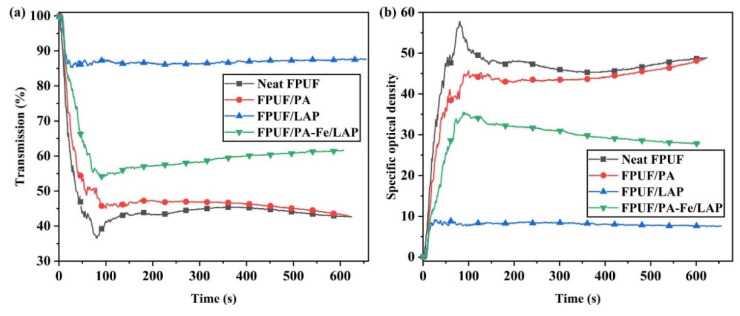
Light transmission (**a**) and specific optical density (**b**) curves of neat FPUF, FPUF/PA, FPUF/LAP, and FPUF/PA-Fe/LAP by SDT.

The smoke density test (SDT) was used to further study the smoke suppression performance of the flame-retardant coating, and the results are shown in Figure 9. After neat FPUF was ignited, the smoke was released rapidly, and the visibility is reduced sharply. At 81 s, the light transmission of neat FPUF is reduced to 36.5% and the smoke density (D_s_) is 57.8. Flame-retardant FPUFs can inhibit smoke release and improve the chance of escape in a fire. It is worth noting that the maximum smoke density (D_s,max_) of FPUF/LAP is 84.1% lower than that of neat FPUF, which can be attributed to the covering effect of inorganic particle LAP. LAP breaks down into highly inorganic thermal stable compounds and silica, which in turn convert to glass on the FPUF surface, preventing volatiles from leaving the condensed phase [53]. However, the smoke suppression effect of FPUF/PA is poor. When LAP and PA-Fe act together, they still have good smoke suppression performance. The D_s,max_ of FPUF/PA-Fe/LAP is decreased by 38.5% in contrast to neat FPUF.

### 2.4. Mechanical Properties

**Figure 10:**
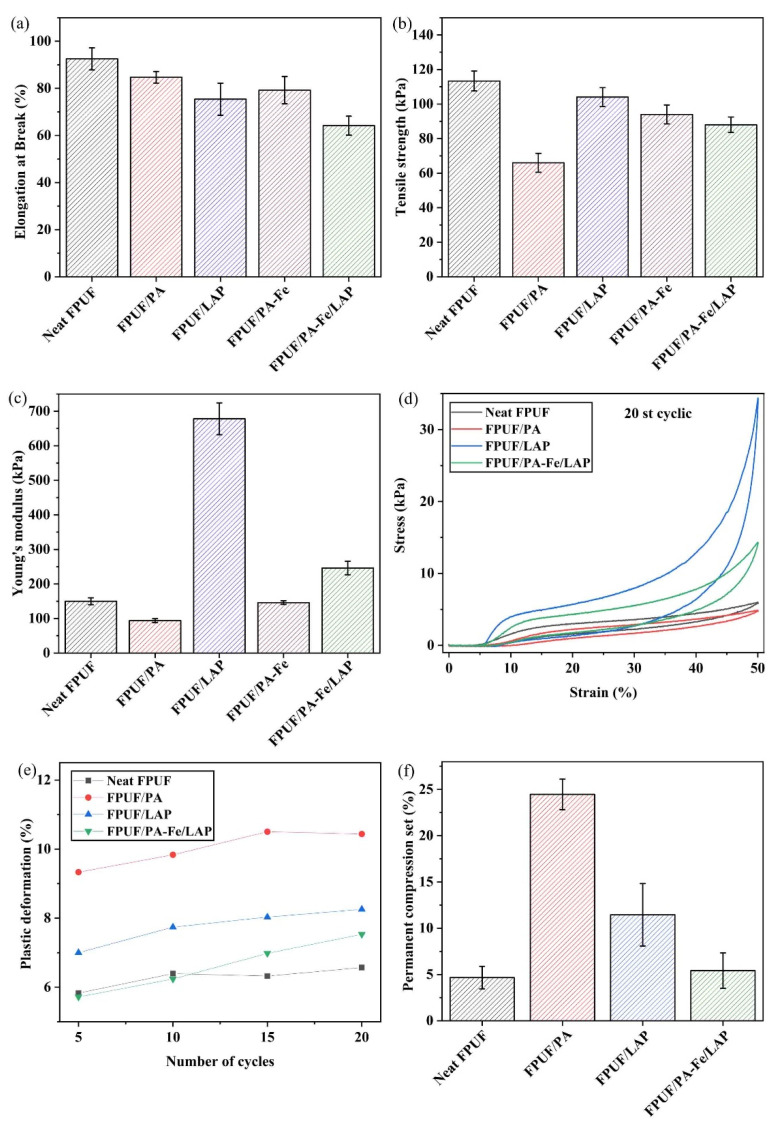
Tensile properties (**a**–**c**), stress-strain (**d**,**e**), and permanent deformation (**f**) of neat FPUF, FPUF/PA, FPUF/LAP, and FPUF/PA-Fe/LAP.

The mechanical properties are also one of important parameters in the application of materials, especially the tensile properties, resilience, and compression resistance to permanent deformation. Therefore, the results are shown in Figure 10a–f. After flame retardant treatment, the elongation at break is decreased, especially the elongation at break of FPUF/PA-Fe/LAP is deteriorated by 30.6% compared with neat FPUF (Figure 10a). This may be due to the existence of PA-Fe/LAP coating limiting the movement of molecular segments in FPUF. Similarly, the tensile strength of flame-retardant FPUF is also deteriorated (Figure 10b), which may be attributed to the excessive acidity of PA damaging FPUF or the stress concentration of agglomerated LAP [54]. However, after coating LAP and PA-Fe/LAP, the Young’s modulus is increased, which can be due to the rigid film formed by the coating on the surface of FPUF.

After 20 cyclic compressing-releasing test at 50% compression strain, both neat FPUF and flame-retardant FPUFs can return to the original shape, and the height loss of flame-retardant FPUFs is smaller (FPUF/PA loses 10.44%, FPUF/LAP loses 8.26%, and FPUF/PA-Fe/LAP loses 7.53%) (Figure 10d,e), showing good cyclic compression. Interestingly, when PA-Fe and LAP are combined, the height loss is less than that of LAP or PA alone.

Moreover, 50% compression resistance to permanent deformation of FPUF was also measured (Figure 10f). The deformation of neat FPUF is only 4.66%, while the deformation of FPUF/PA increases sharply. Besides, the deformation of FPUF/LAP (11.45%) also increases, which may be due to the rigid material of LAP coating and hinders the rebound of FPUF/LAP. Fortunately, when the flame-retardant coating contains PA-Fe and LAP, the deformation can be significantly reduced, and the deformation is only 5.43%, which is similar to that of neat FPUF.

Overall, FPUF/PA with the best effect of heat suppression has significantly deteriorated the mechanical properties. However, the elongation at break of FPUF/PA-Fe/LAP deteriorated slightly, and the effects of other mechanical properties were within an acceptable range. Therefore, PA-Fe/LAP coating is more suitable as the flame retardant and antidripping coating for FPUF.

## 3. Materials and Methods

### 3.1. Materials

PA (50 wt.% aqueous solution) was supplied by Shanghai Macklin Biochemical Co., Ltd. (Shanghai, China). Fe_2_(SO_4_)_3_·*x*H_2_O was produced by Sinopharm Chemical Reagent Co., Ltd. (Shanghai, China). LAP was purchased from Shandong Yousuo Huagong Co., Ltd. (Shandong, China). FPUF (FM28) with a density of 27.8 ± 2 kg/m^3^ was obtained from Hangzhou Guangsheng Foam Plastics Co., Ltd. (Hangzhou, China). Deionized water was made in the laboratory.

### 3.2. Preparation of Flame-Retardant FPUFs

Firstly, LAP, PA, and Fe_2_(SO_4_)_3_·*x*H_2_O were dissolved in deionized water and stirred at room temperature to prepare these flame retardants according to a certain molar ratio (1:0:0, 0:1:0, 25:1:135, and 0:1:135, named LAP, PA, PA-Fe/LAP, and PA-Fe, respectively). Secondly, FPUF was cut into the desired size, and dried at 80 °C. Afterward, FPUF was placed in the above flame-retardant solutions separately. They were pressed continuously to fully coat the FPUF surface with the flame retardants. Finally, the flame-retardant FPUFs were dried at 80 °C until a constant weight was obtained. The obtained flame-retardant FPUFs were named FPUF/PA, FPUF/LAP, FPUF/PA-Fe, and FPUF/PA-Fe/LAP according to the flame-retardant solutions.

### 3.3. Characterization

#### 3.3.1. The Micromorphology and Element Distribution

The morphology of neat FPUF, flame-retardant FPUFs, and char residues after CCT was obtained on a scanning electron microscope (TESCAN-VEGA3, Brno, Czech Republic). Additionally, the element composition of flame-retardant FPUFs was measured by an energy dispersive X-ray analyzer (XSAM80, Kratos Co, Manchester, UK). The samples were sputter-coated by a thin gold layer before the test.

#### 3.3.2. Thermal Stability

The thermo-oxidative stability was assessed through thermogravimetric analyses carried out with a with a Perkin-Elmer STA 6000 TG analyzer ((Perkin-Elmer Ltd., Waltham, MA, USA). The test was obtained under an air flow of 30 mL·min^−1^ and the heating rate was set at 10 °C·min^−1^ from 40 to 800 °C. The thermal stabilities and the consist of gaseous products were performed using a TG analyzer with a Perkin-Elmer FTIR spectrometer (Perkin-Elmer Ltd., Waltham, MA, USA). The samples were heated under 50 mL·min^−1^ flow of nitrogen, and other conditions were the same as in air.

#### 3.3.3. Flame Retardancy and Combustion Behaviors

UL-94 was performed on an LFY-601A vertical flame tester (Shandong Textile Academy, Qingdao, China) with the sample size of 100 × 50 × 15 mm^3^. UL-94 was measured in the light of the ASTM D 3801. LOI, which sample size was 150 × 10 × 10 mm^3^, was obtained on an LFY-606 B oxygen index meter (Shandong Institute of Textile Science, Qingdao, China) in accordance with the GB/T2406.2-2008 standard. Burning behaviors were collected using a cone calorimeter (Fire Testing Technology, West Sussex, UK) according to the ISO 5660-1 standard. The sample had dimensions of 100 × 100 × 25 mm^3^ and was exposed to 35 kW·m^−2^ heat flux. SDT was executed by a JCY-2 equipment (SPT Co., Yangzhou, China) following the EN ISO 5659-2. The sample size was set to be 75 × 75 × 25 mm^3^ and under a pilot flame of 25 kW·m^−2^.

#### 3.3.4. Mechanical Properties and Air Permeability

The tensile strength was evaluated by a universal testing machine (INSTRON 5967, INSTRON, Norwood, MA, USA) referenced from the GB/T 6344-2008. The sample was tailored into 40 × 10 × 10 mm^3^ at a crosshead speed of 500 mm·min^−1^. The resilience of the samples was performed using a universal testing machine (INSTRON 5965, INSTRON, Norwood, MA, USA) that was interfaced to a 5000 N load cell. The samples were subjected to 20 cyclic compressing-releasing tests at 50% compression strain at a compression rate of 20 mm·min^−1^. The dimension of each sample was 20 × 20 × 20 mm^3^. The 50% compression test was measured following the ISO1856-2000. The sample was tailored into 50 × 50 × 10 mm^3^ and compressed by two metal plates for 22 h at 77 °C. The air permeability of FPUFs was measured by a fully automatu permeability instrument (YG461E-III, Ningbo Textile Instrument Factory, Ningbo, China) at 100 Pa.

## 4. Conclusions

PA-Fe/LAP coating was constructed on the FPUF surface by dip-coating, which gives FPUF flame retardancy and antidripping without deteriorating its cyclic compression and permanent deformation. FPUF/PA-Fe/LAP has flame retardancy and reduces the risk of a secondary fire, as evidenced by a satisfying UL-94 V-0 rating and a higher LOI value of 24.5%, and inhibits heat and smoke release. The PHRR and D_s,max_ values of FPUF/PA-Fe/LAP are reduced by 38.7% and 38.5% compared with those of neat FPUF. Moreover, although the elongation at break of FPUF/PA-Fe/LAP reduces by 30.6% compared with neat FPUF, the height loss of FPUF/PA-Fe/LAP (from 6.57% to 7.53%) and the deformation (from 4.66% to 5.43%) are close to those of neat FPUF. Therefore, the deterioration of PA-Fe/LAP coating on the mechanical properties of FPUF is acceptable. This work provides an effective way to enhance the flame retardancy of FPUF.

## Figures and Tables

**Table 1:** Summary of TG data for neat FPUF, FPUF/PA, FPUF/LAP, and FPUF/PA-Fe/LAP in N_2_.

Sample	T_5%_ (°C)	T_max1_ (°C)	R_max1_ (%/min)	T_max2_ (°C)	R_max2_ (%/min)	Residues at 800 °C (%)
Neat FPUF	258	291	6.4	374	14.3	9.7
FPUF/PA	169	264	2.6	325	9.1	29.9
FPUF/LAP	260	289	4.1	371	8.3	33.3
FPUF/PA-Fe/LAP	226	286	4.4	380	10.2	19.3

**Table 2:** Summary of TG data for neat FPUF, FPUF/PA, FPUF/LAP, and FPUF/PA-Fe/LAP in air.

Sample	T_5%_ (°C)	T_max1_ (°C)	R_max1_ (%/min)	T_max2_ (°C)	R_max2_ (%/min)	Residues at 800 °C (%)
Neat FPUF	251	276	11.4	330	11.2	11.1
FPUF/PA	196	256	3	320	11.8	27.1
FPUF/LAP	256	297	13.5	-	-	33.1
FPUF/PA-Fe/LAP	231	300	13.7	-	-	22.4

**Table 3:** The detailed data of UL-94 and LOI.

	Weight Gain (wt.%)	t_1_ (s)	t_2_ (s)	Burning to the Fixture	Dripping	UL-94	LOI (%)
Neat FPUF	0.0 ± 0.0	>30	-	Yes	Yes	N.R.	17.0
FPUF/PA	43.8 ± 1.6	1 ± 1	3 ± 1	Yes	No	N.R.	29.9
FPUF/LAP	37.3 ± 0.2	>30	-	Yes	No	N.R.	19.6
FPUF/PA-Fe	40.2 ± 0.9	>30	-	Yes	No	N.R.	20.7
FPUF/PA-Fe/LAP	41.9 ± 0.7	0 ± 0	1 ± 1	No	No	V-0	24.5

**Table 4:** The detailed data from CCT.

Sample	TTI (s)	PHRR (kW/m^2^)	T_PHRR_ (s)	THR (MJ/m^2^)	FIGRA kW/(m^2^·s)	PSPR (m^2^/s)	TSP (m^2^)	PSEA (m^2^/kg)	CO/CO_2_	Residues (wt.%)
Neat FPUF	3	419	40	19	10.5	0.05	4.5	4385	0.0134	17.2
FPUF/PA	5	126	25	13	5.0	0.03	2.7	4012	0.0581	32.7
FPUF/LAP	6	190	20	14	9.5	0.02	1.5	3321	0.0923	42.1
FPUF/PA-Fe/LAP	5	257	50	17	5.1	0.03	3.0	3371	0.0171	23.1

## Data Availability

The data are available upon request.
